# Solitary large hepatic metastasis in an elderly patient with anaplastic thyroid carcinoma: a case report

**DOI:** 10.1186/1757-1626-2-41

**Published:** 2009-01-12

**Authors:** Alexandra Adamidou, Panagiotis Kotsaftis, Maria Vlachou, Afroditi Haritanti, Ioannis Xanthakis, Anestis Zantidis, George Ntaios

**Affiliations:** 1 First Propedeutic Department of Internal Medicine, AHEPA Hospital, Aristotle University, Thessaloniki, Greece, S. Kiriakidi 1, AHEPA Hospital, 54636, Thessaloniki, Greece

## Abstract

**Background:**

We present the case of a patient who presented with anaplastic thyroid carcinoma with a solitary large liver metastasis. Hepatic metastases are extremely rare in anaplastic thyroid carcinoma.

**Case presentation:**

We report the case of a 78-year old Greek man who presented with voice hoarseness, dyspnoea and a large mass on the anterior surface of the cervical region (neck), as well as constitutional symptoms such as anorexia, weight loss and malaise. On physical examination, the thyroid was hard and tender on palpation and a liver mass was palpable. Routine biochemistry yielded normal results except increased C-reactive protein, alkaline phosphatase and γ-glutaryl transferase. Other biochemical tests including tumor markers, thyroid hormones, antithyroid antibodies, hepatitis B and hepatitis C antibodies were negative. Imaging methods revealed enlargement of the left thyroid lobe extending to the anterior mediastinum and compressing the trachea, metastatic bilateral pulmonary lesions and a large, nodular, contrast-enhanced mass-occupying lesion in the right hepatic lobe. The findings of fine-needle aspiration biopsy of the thyroid were consistent with anaplastic carcinoma. Liver biopsy showed infiltrations by poorly differentiated anaplastic cells, few of which were slightly positive for thyroglobulin. These findings were suggestive of a hepatic metastasis originating from anaplastic thyroid carcinoma. During his hospitalization, the patient suffered progressive obstruction of the trachea due to rapid increase of the thyroid gland mass. The tumor was considered unresectable due to its advanced stage, as well as the presence of extrathyroid metastatic lesions. The patient was irradiated on cervical region (neck) and upper mediastinum with a daily dose of 300 cGy for 10 days which resulted in mild improvement of obstructive phenomena. However, his clinical condition deteriorated rapidly and he died within a few days.

**Conclusion:**

Solitary, large hepatic metastasis may constitute a rare complication in anaplastic thyroid carcinoma.

## Background

Anaplastic thyroid carcinoma is a rare, highly aggressive undifferentiated tumor. In most cases, regional or distant expansion is already present at diagnosis. Distant metastases usually involve lungs and rarely, bones, mediastinum and brain [1]. On the contrary, hepatic metastases are extremely rare [2,3]. In this paper, we present the case of a patient who presented with anaplastic thyroid carcinoma with pulmonary and hepatic metastases.

## Case presentation

**Figure 1:**
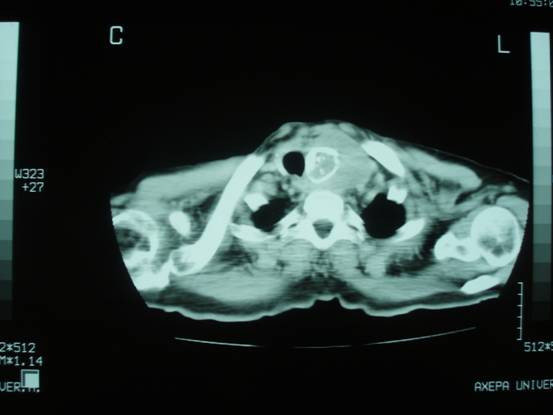
**Computed tomography scan of the cervical neck**.

**Figure 2:**
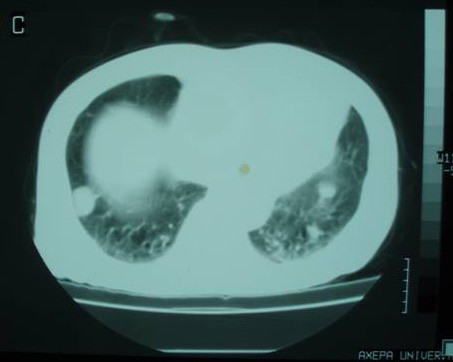
**Computed tomography scan of the thorax**.

**Figure 3:**
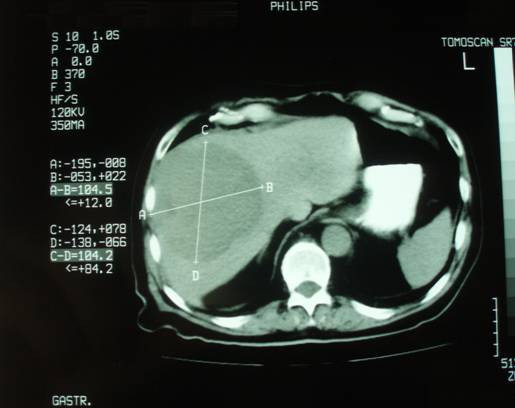
**Computed tomography scan of the liver**.

We report the case of a 78-year old Greek man who presented with voice hoarseness, dyspnoea and a large mass on the anterior surface of the cervical region as well as constitutional symptoms such as anorexia, weight loss and malaise. On physical examination, the thyroid was hard and tender on palpation and a liver mass was palpable. Routine biochemistry yielded normal results except increased C-reactive protein, alkaline phosphatase and γ-glutaryl transferase. Other biochemical tests including tumor markers, thyroid hormones, antithyroid antibodies, hepatitis B (anti-HBV) and hepatitis C (anti-HCV) antibodies were negative. Chest X-ray showed enlargement of the superior mediastinum, deviation of the trachea to the left and bilateral pulmonary metastatic lesions. Computed tomography scan of the neck, thorax and abdomen revealed enlargement of the left thyroid lobe extending to the anterior mediastinum and compressing the trachea, metastatic bilateral pulmonary lesions and a large, nodular, contrast-enhanced mass-occupying lesion in the right hepatic lobe (figures 1, 2, 3).

**Figure 4:**
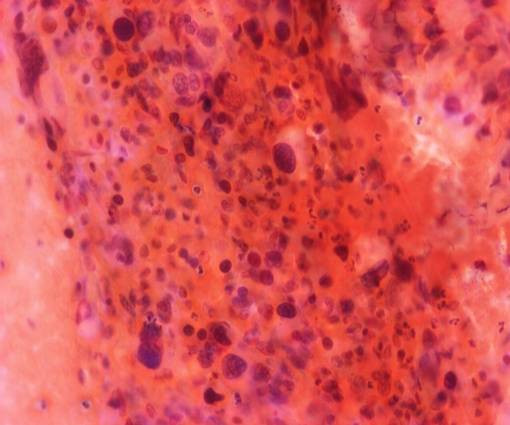
**Fine-needle aspiration biopsy smear of the thyroid (Papanicolaou stain)**.

**Figure 5:**
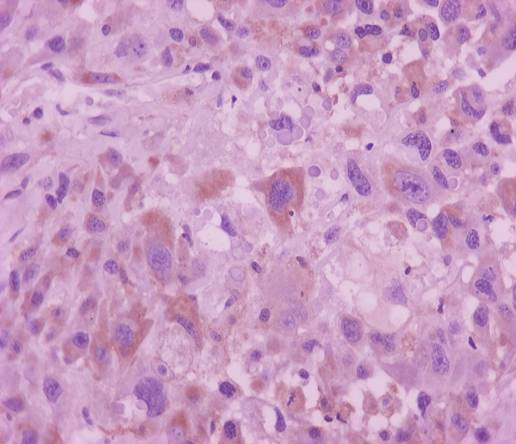
**Liver biopsy stained with thyroglobulin**.

Fine-needle aspiration biopsy of the thyroid was performed. The smear was very cellular with extremely pleomorphic cells in size-small to giant forms-and in shape-round, oval and spindle. These cells were isolated and in tissue fragments with large irregular nuclei, which showed clumped chromatin with parachromatin clearing and occasional large, multiple and irregular nucleoli (figure 4). The immunocytochemical staining revealed that the neoplasmatic cells were negative for calcitonin and neuron specific enolase and a few of them were slightly positive for thyroglobulin. Hence, the diagnosis of anaplastic thyroid carcinoma was reached. Liver biopsy showed infiltrations with poorly differentiated anaplastic cells. These anaplastic cells were morphologically similar with the cells of the fine-needle aspiration specimen. Moreover, immunocytochemical staining revealed that a small number of these anaplastic cells were slightly positive for thyroglobulin (figure 5). Furthermore, immunocytochemistry yielded neoplasmatic cells positive for cytokeratin-7 and negative for cytokeratin-20, carcinoembryogenic antigen (CEA), carbohydrate antigen 19-9 (CA 19-9) and hepatocyte paraffin-1 (HepPar-1). These findings were suggestive of hepatic metastasis from the primary anaplastic thyroid carcinoma.

During his hospitalization, the patient suffered progressive obstruction of the trachea due to rapid increase of the thyroid gland mass. The tumor was considered unresectable due to its advanced stage, as well as the presence of extrathyroid metastatic lesions. The patient was irradiated on cervical region and upper mediastinum with a daily dose of 300 cGy for 10 days which resulted in mild improvement of obstructive phenomena. However, the patient soon deteriorated with thrombocytopenia, elevated d-dimers, prolonged prothrombin time, anemia and transaminasaemia due to disseminated intravascular coagulation and he died within a few days.

## Conclusion

This case shows that although rare, solitary large hepatic metastasis may constitute a complication in anaplastic thyroid carcinoma. Usually, the differential diagnosis of a metastatic hepatic lesion does not include the thyroid gland among the first organs to be investigated, since the thyroid gland usually metastasizes in lungs, bones and brain. From this aspect, the thorough physical examination of the thyroid is essential since in case of positive findings, the clinical suspicion of anaplastic thyroid carcinoma should be raised.

## Abbreviations

anti-HBV: hepatitis B antibodies; anti-HCV: hepatitis C antibodies; CEA: carcinoembryogenic antigen; CA 19-9: carbohydrate antigen 19-9; HepPar-1: hepatocyte paraffin-1.

## Consent

Written informed consent was obtained from the wife of the patient for publication of this case report and accompanying images. A copy of the written consent is available for review by the Editor-in-Chief of this journal.

## Competing interests

The authors declare that they have no competing interests.

## Authors' contributions

AA drafted the manuscript. PK and GN both wrote the manuscript. MV made the pathological diagnosis. AH performed liver biopsy. IX and AZ both participated in the care of the patient and interpretation of the testing. All authors read and approved the final manuscript.
